# Can early postoperative ultrasound replace routinary flexible laryngoscopy after neuromonitoring-assisted thyroid surgery?

**DOI:** 10.1007/s13304-025-02199-w

**Published:** 2025-04-07

**Authors:** Luca Sessa, Andrea Attard, Francesco Cupido, Stefania Marchisotta, Adele Maniglia, Francesco Pennestrì, Carmela De Crea, Marco Raffaelli

**Affiliations:** 1 Division of Endocrine Surgery, Fondazione Istituto G. Giglio, 90015 Cefalù, PA Italy; 2 Università Unicamillus, Saint Camillus International University of Health Sciences, Rome, Italy; 3 Centro Clinico di Endocrinologia “Marco Attard”, 90143 Palermo, Italy; 4 Division of Endocrine and Metabolic Surgery, Fondazione Policlinico Universitario “Agostino Gemelli”, IRCCS, L.go A. Gemelli 8, 00168 Rome, Italy; 5 Centro di Ricerca in Chirurgia delle Ghiandole Endocrine e dell’Obesità, Università Cattolica del Sacro Cuore, 00168 Rome, Italy; 6 Division of Endocrine Surgery, Fatebenefratelli Isola Tiberina, Gemelli Isola, 00186 Rome, Italy

**Keywords:** Laryngeal ultrasound, Intraoperative neuromonitoring, Flexible laryngoscopy, Laryngeal palsy, Postoperative complications

## Abstract

Ultrasound (US) has been proposed to assess vocal cord motility after thyroid surgery since early post-operative flexible laryngoscopy (FL) is not readily available in all centers. We aimed to verify if FL can be avoided in intraoperative neuromonitoring (IONM)-assisted thyroid surgery followed by early US vocal cord motility evaluation. Two hundred and thirty-four patients who underwent IONM-assisted thyroidectomy were included. When total thyroidectomy (TT) was planned, the surgical procedure was stopped in case of loss of signal (LOS) or significant signal reduction (SSR) after the dissection of the first lobe. US vocal cord motility evaluation and FL were performed in all patients on postoperative day 1. Among 377 nerves at risk (91 thyroid lobectomies and 143 TT), post-operative FL showed 9 unilateral vocal cord palsies and 4 unilateral hypomotilities. IONM results showed 15 LOS and 10 SSR. US vocal cord motility evaluation confirmed unilateral vocal cord palsy in 8 cases and correctly identified normal post-operative vocal cord motility in 13 patients with altered IONM results. FL was able to diagnose 4 unilateral vocal cord hypomotilities in patients with normal IONM results and US evaluation. Overall accuracy was 91.4% for IONM and 96.5% for US, respectively. Early postoperative US evaluation after IONM-assisted thyroid surgery improves the overall accuracy of IONM alone in assessing laryngeal function after thyroid surgery. Nonetheless, IONM results and post-operative US do not replace FL, which remains the gold standard for early detection of laryngeal motility changes also in asymptomatic patients.

## Introduction

Post-thyroidectomy transient or permanent inferior laryngeal nerve (ILN) injury is one of the most feared complications and one of the leading causes of medicolegal litigation against surgeons [1–4]. Moreover, bilateral ILN palsy has been described as a “serious surgical calamity” because of the risk of acute respiratory failure potentially requiring re-intubation and, in some cases, tracheotomy [1, 5, 6].

Unfortunately, the deep applied knowledge of the anatomy and a meticulous surgical technique preserving the anatomical integrity of the ILN are not enough to ensure a normal post-operative laryngeal function [1]. In the last decades, intraoperative neuromonitoring (IONM) has been introduced as a tool to obtain intraoperative information about the functional integrity of the ILNs prior to, during and after the surgical dissection [1, 2, 7–12]. To date, there is no high-level evidence supporting the use of IONM to reduce unilateral ILN both for intermittent and continuous IONM, but its role in the prevention of bilateral laryngeal palsy is well established [10–13]. Moreover, the IONM role also depends on the surgical team’s decision-making based on the neurophysiological information provided by the system [10, 11]. IONM-assisted thyroid surgery implies the use of modern technology, and several variables potentially can cause inaccurate responses (due to factors related to the patients, the anesthesiologists, the surgeons, and the IONM machines) [1, 14, 15]. Inaccurate findings are reported in 3.8–23% of IONM-assisted procedures [14, 15]. For these reasons, flexible laryngoscopy (FL) remains the gold standard to assess vocal cord motility after thyroid surgery since the meticulous ILN dissection preserving the anatomic integrity, IONM reports, and subjective voice evaluation are not sufficient to unequivocally post-operatively assess laryngeal motility [1, 16, 17].

Since early postoperative FL is not readily available in all centres, Ultrasound (US) has been proposed as an easy-to-perform, unexpansive, reproducible, noninvasive, non-aerosol-generating procedure to assess vocal cord motility after thyroid surgery [15, 17–32].

We aimed to verify whether FL can be avoided in IONM-assisted thyroid surgery, followed by an early postoperative US vocal cord motility evaluation.

## Methods

### Study design

All patients who underwent primary thyroid surgery at the Division of Endocrine Surgery of the Fondazione Istituto G. Giglio in Cefalù (Palermo, Sicily, Italy) were included between December 2022 and November 2023. Demographic, clinical, operative, pathological, and follow-up data were prospectively registered in a specifically designed database.

Exclusion criteria were: previous neck surgery; pre-operative evidence of laryngeal palsy; patients undergoing parathyroid surgery alone; procedures not requiring ILN dissection (i.e. lateral neck dissection after previous thyroidectomy).

Indications for surgery were based on institutional integrated care pathways and national and international guidelines [16, 33].

All patients underwent pre-operative FL. All the procedures were performed by two expert endocrine surgeons (LS and AA), both with documented extensive experience in thyroid surgery and IONM use. All the procedures were intermittent IONM-assisted. Pre- and post-dissection vagal and ILN signals were recorded by surgeons in all cases. Intermittent-IONM system (C2 Xplore® system—Inomed Medizintechnik GmbH, Emmendingen, Germany) was used in all the cases. C2 Xplore® system (Inomed Medizintechnik GmbH, Emmendingen, Germany) equipment for intermittend-IONM of the ILN consists of: laryngeal electrode Select (sticker), subdermal needle electrode, stimulation probe, C2 Xplore system®, electrode connection box, connecting cable, and mute sensor. When bilateral ILN dissection was planned, the surgical procedure was stopped after the dissection of the first side in case of loss of signal (LOS) or significant (≥ 50%) signal reduction (SSR). All patients in which a TT was planned had been previously informed about the possibility of a staged thyroidectomy in case of LOS [8, 34].

In case of LOS, a troubleshooting algorithm has been applied to exclude a system malfunction: the presence of a laryngeal twitch, electromyography response from contralateral vagal nerve stimulation, evaluation for monitoring equipment dysfunction (interface-connector box), evaluation for incorrect use of neuromuscular blocking agents, and malposition of recording electrodes (e.g., electromyography tube) [1, 2].

On postoperative day 1, US vocal cord motility evaluation was performed using a multifrequency linear probe with the patient in a supine position and mild neck hyperextension. In patients with unsatisfactory midline views, an oblique lateral approach was performed. A single operator performed all the first-day postoperative US evaluations. This same professional was part of the surgical team in all the operations and was therefore aware of the IONM data and of the surgical details. He has specific ultrasound training, having obtained the diploma in ultrasound for endocrine neck diseases. A Samsung® HM70EVO US machine was used for all the post-operative laryngeal evaluations. A single multifrequency linear probe with a frequency range of 3–16 MHz was used. The initial settings are directly configured by the device for small parts and thyroid imaging. Lower frequencies were used for better visualization of the vocal cords and their movements, especially in patients with thick thyroid cartilage. In all cases, both frontal and lateral scans were performed to assess the simultaneous movement of the arytenoids. Laryngeal movements were evaluated during spontaneous breathing, apnea, and phonation. Vocal cord paralysis is defined as the absence of movement during ultrasound evaluation of the vocal cord in spontaneous breathing and phonation. In cases where proper ultrasound visualization of the glottic plane is not possible, paralysis is defined as the absence of movement of the corresponding arytenoid cartilage.

On postoperative day 1, FL was performed on all patients. All the FL was performed by an expert ENT with extensive documented experience in FL. In all the cases a 2.8 mm high definition digital flexible videolaryngoscope was used (Mindsion®) connected to a Monitor VS100 (1280 × 800), Mindsion®. The instrument is inserted through the lower nasal passage. The nasopharynx, the base of the tongue, the epiglottis, and subsequently the false vocal cords and true vocal cords are examined, both morphologically and functionally. The patient is asked to phonate, pronouncing the target vowel "i". Additionally, the subglottic space and the laryngotracheal axis are observed.

The postoperative protocol adopted for the management of hypoparathyroidism was described elsewhere [35].

Pathologic tumour staging was defined in thyroid malignancy according to the 2017 8th edition of the American Joint Committee on Cancer pTNM staging system [36].

### Definitions

Thyroid lobectomy (TL) was defined as the extracapsular removal of one thyroid lobe with the isthmus, while total thyroidectomy (TT) was defined as total, bilateral extracapsular thyroid removal.

Unilateral central neck dissection (CND) included prelaryngeal, pretracheal, and paratracheal nodes on the side of the tumour (paratracheal nodes contralateral to the tumour were not included) [37]. Bilateral CND included the removal of prelaryngeal, pretracheal, and both the left and right paratracheal nodes [37]. Lateral neck dissection was performed in all cases with therapeutic intent. Comprehensive lateral neck dissection was defined as compartment-oriented functional lateral neck dissection, including levels II, III, IV, and V [38].

LOS is defined as electromyography signal degrades to < 100 µV from initial satisfactory electromyography with suprathreshold stimulation level (i.e., > 1 or 2 mA) in a dry field and no laryngeal twitch. LOS could be classified as Type 1 (segmental) and Type 2 (global) [1, 2].

SSR was defined as a post-dissection signal reduction ≥ 50% when compared to the pre-dissection recorded value.

Inaccurate IONM results were defined as discordance between IONM reports and postoperative FL.

Inaccurate postoperative US laryngeal evaluation was defined as the discordance between US and postoperative FL.

Postoperative complications (namely hypoparathyroidism and ILN palsy) were defined as permanent in case of persistence of serum PTH level below the normal range and persistence of ILN palsy at FL 6 months after the operation since follow up data were obtained 6 months after surgery.

### Study end-point

The primary endpoint was to verify if postoperative FL can be avoided in IONM-assisted thyroid surgery, followed by early postoperative US vocal cord motility evaluation.

The secondary endpoint was to assess preoperative risk factors for inaccurate IONM and postoperative laryngeal US results.

### Statistical analysis

Categorical or dichotomous variables were summarised using absolute numbers (n) with percentages (%) and were compared using the Chi-squared test. Normal distribution was assessed using the Shapiro-Wilks test. Continuous variables were summarised using means (m) and standard deviations (SD). We used paired sample t-test or Wilcoxon test to compare continuous variables, depending on data distribution of the analyzed population. IONM results and postoperative US vocal cord motility evaluation were compared against postoperative FL findings. Sensitivity, specificity, positive predictive value, negative predictive value, and overall accuracy for each method were calculated (95% confidence interval). All statistical analyses were performed at the significance level P-value (P) = 0.05. All analyses were conducted using Stata version 16.0 (StataCorp, College Station, Texas 77,845 USA).

## Results

Overall, 234 patients were included (59 males and 175 females with a mean age of 53 years and a mean Body Mass Index -BMI- of 26.2 kg/m^2^). TT was performed in 143 (61.1%) cases and a TL in the remaining 91 (38.9%) cases. A concomitant CND was performed in 17 cases (7.3%), unilateral in 4 and bilateral in the remaining 13 cases, a LND was accomplished in 5 cases (2.1%). Final histology showed malignant disease in 55 cases (23.5%): 49 papillary thyroid carcinomas (21 pT1a, multifocal in 4 cases, 22 pT1b, multifocal in 7 cases, 5 pT2 and 1 pT4; pN1a in 10 cases and pN1b in 5 cases), 4 follicular thyroid carcinomas (1 pT1b, 2 pT2 and 1 pT3a) and 2 medullary thyroid carcinomas (2 pT1am, 1 pN1a). Regarding post-operative complications, a post-operative hematoma requiring reoperation occurred in 3 patients (1.3%), while a transient hypoparathyroidism was registered in 47 patients (20%) and a definitive hypoparathyroidism in 5 cases (2.1%). Overall, 11 patients (4.7%) showed transient unilateral laryngeal palsy, and 2 patients (0.9%) showed definitive unilateral laryngeal palsy. No bilateral ILN palsies occurred.

Table 1 reports the demographic, operative, pathological, and follow-up characteristics of all the included patients.

**Table 1:** Demographic, operative, pathological and follow-up characteristics of the included patients

Patients	234
Age (± SD^a^) (range) years	53 ± 14 (17–81)
Male/female	59 (25.2%)/ 175 (74.8%)
BMI^b^ (± SD^a^) (range) Kg/m^2^	26.2 ± 5.1 (15–45)
BMI^b^ ≥ 25 kg/m^2^ y/n	145 (62%)/89 (38%)
BMI^b^ ≥ 30 kg/m^2^ y/n	50 (21.4%)/184 (78.6%)
Smokers y/n	66 (28.2%)/168 (71.8%)
ASA^c^ score 1/2/3	20 (8.5%)/ 146 (62.4%)/68 (29.1%)
Mallampati score 1/2/3/4	70 (30%)/82 (35%)/ 65 (27.8%)/ 17 (7.2%)
Hyperthyroidism y/n	44 (18.8%)/ 190 (81.2%)
TT^d^/TL^e^	143 (61.1%)/91 (38.9%)
CND^f^ y/n	17 (7.3%)/217 (92.7%)
Extension cervical/Cervico-mediastinal	204 (87.2%)/ 30 (12.8%)
Final histology benign/malignant disease	179 (76.5%)/55 (23.5%)
Post-operative hematoma y/n	3 (1.3%)/231 (98.7%)
Transient hypoparathyroidism y/n Definitive hypoparathyroidism y/n	47 (20%)/187 (80%) 5 (2.1%)/229 (97.9%)
Transient laryngeal nerve palsy y/n	11 (4.7%)/223 (95.3%)
Definitive laryngeal nerve palsy y/n	2 (0.9%)/232 (99.1%)

^a^Standard deviation

^b^Body mass index

^c^American Society of Anesthesiologists

^d^Total thyroidectomy

^e^Thyroid lobectomy

^f^Central neck dissection

Overall, IONM results showed a normal response in 209 (89.3%), SSR in 10 (4.3%) and global LOS in 15 (6.4%) patients, respectively. US vocal cord motility evaluation on post-operative day 1 showed no visible vocal cord at the US in 7 cases (3%) and, among the remaining patients, 219 normal vocal cord motility (96.5%) and 8 unilateral vocal cord paralysis (3.5%). Post-operative FL showed normal motility in 221 cases (94.5%), unilateral hypomotility in 4 (1.7%) and unilateral paralysis in 9 (3.8%) cases, respectively (Table 2).

**Table 2:** Laryngeal evaluations of the included patients

Patients	234
Nerves at risk	377
Post-operative flexible laryngoscopy	
Normal	221 (94.5%)
Hypomotility/paralysis	4 (1.7%)/9 (3.8%)
IONM^a^ results	
Normal	209 (89.3%)
SSR^b^/LOS^c^	10 (4.3%)/15 (6.4%)
Post-operative laryngeal US^d^	
VC^e^ visible/not visible	227 (97%)/7 (3%)
Normal/Paralysis	219 (96.5%)/8 (3.5%)

^a^Intraoperative neuromonitoring

^b^Significant (≥ 50%) signal reduction

^c^Loss of signal

^d^Ultrasound

^e^Vocal cords

The sensitivity, specificity, and overall accuracy for IONM results and post-operative US were 69.2% (42.4–87.3%), 92.8% (88.6–95.5%), 91.4%, (87.2–94.4%) 61.5% (38.6–81.2%), 100%, and 96.6% (95.1–99.0%), respectively (Tables 3 and 4).

**Table 3:** Intraoperative neuromonitoring results analysis

Intraoperative neuromonitoring	
True positive	9
True negative	205
False positive	16
False negative	4
Sensitivity	69.2%
Specificity	92.8%
Positive predictive value	36%
Negative predictive value	98%
Overall accuracy	91.4%

**Table 4:** Post-operative laryngeal ultrasound results analysis

Laryngeal Ultrasound	
True positive	8
True negative	218
False positive	0
False negative	5
Sensitivity	61.5%
Specificity	100%
Positive predictive value	100%
Negative predictive value	97.7%
Overall accuracy	96.6%

Among 150 planned TT, the procedure was stopped after dissection of the first side in 7 cases (4.7%, 3 SSR and 4 LOS). FL showed normal vocal cord motility in 2 out of 3 SSR and 2 out of 4 LOS, respectively. Two patients required completion thyroidectomy (respectively for Basedow disease and papillary thyroid carcinoma pT1bmN1a); the other five patients have chosen to wait and see follow-up).

Among 16 patients with registered post-operative dysphonia, in 4 (25%) cases FL showed normal vocal cord motility. Moreover, in one patient without dysphonia, post-operative FL showed unilateral vocal cord hypomotility.

Among pre-operative parameters, a higher Mallampati score is a risk factor for inaccurate IONM results and inaccurate post-operative laryngeal US (Tables 5 and 6).

**Table 5:** Risk factors for inaccurate Intraoperative Neuromonitoring (IONM) results

	Accurate IONM	Inaccurate IONM	*p*
Patients	214	20	NS*
Age (± SD^a^) (range) years	53 ± 14.2 (75–81)	57 ± 10.1 (36–75)	NS*
Male/Female	54/160	5/15	NS*
BMI^b^ (± SD^a^) (range) Kg/m^2^	26.2 ± 5.1 (15–45)	26.5 ± 4.0 (21–37)	NS*
BMI^b^ ≥ 25 kg/m^2^ y/n	132/82	13/7	NS*
BMI^b^ ≥ 30 kg/m^2^ y/n	45/169	5/15	NS*
Smokers y/n	63/115	3/17	NS*
ASA^c^ score 1/2/3	19/136/59	1/10/9	NS*
Mallampati score 1/2/3/4	66/79/53/16	4/3/12/1	< 0.01
Hyperthyroidism y/n	42/172	2/18	NS*
TT^d^/TL^e^	136/78	7/13	< 0.05
CND^f^ y/n	16/198	1/19	NS*
Extension cervical/cervico-mediastinal	186/28	18/2	NS*
Final histology benign/malignant disease	166/49	14/6	NS*
Post-operative hematoma y/n	2/212	1/19	NS*
Transient Hypoparathyroidism y/n Definitive Hypoparathyroidism y/n	44/170 4/210	3/17 1/19	NS* NS*

^*^Not significant

^a^Standard deviation

^b^Body mass index

^c^American Society of Anesthesiologists

^d^Total thyroidectomy

^e^Thyroid lobectomy

^f^Central neck dissection

**Table 6:** Risk factors for inaccurate post-operative laryngeal ultrasound (US) results

	Accurate us	Inaccurate us	p
Patients	222	5	NS*
Age (± SD^a^) (range) years	53 ± 14.1 (17–81)	57 ± 13.8 (36–72)	NS*
Male/Female	54/168	2/3	NS*
BMI^b^ (± SD^a^) (range) Kg/m^2^	26.2 ± 5.1 (15–45)	25.6 ± 2.9 (22–29)	NS*
BMI^b^ ≥ 25 kg/m^2^ y/n	138/84	3/2	NS*
BMI^b^ ≥ 30 kg/m^2^ y/n	47/175	0/5	NS*
Smokers y/n	63/159	0/5	NS*
ASA score 1/2/3	20/138/64	0/3/2	NS*
Mallampati score 1/2/3/4	69/78/58/17	0/0/5/0	< 0.01
Hyperthyroidism y/n	43/179	0/5	NS*
TT^c^/TL^d^	137/85	4/1	NS*
CND^e^ y/n	17/205	0/5	NS*
Extension cervical/cervico-mediastinal	192/30	5/0	NS*
Final Histology benign/malignant disease	169/53	3/2	NS*
Post-Operative Hematoma y/n	2/220	1/4	< 0.01
Transient Hypoparathyroidism y/n	44/178	2/3	NS*
Definitive Hypoparathyroidism y/n	3/219	1/4	< 0.01

^*^Not significant

^a^Standard deviation

^b^Body mass index

^c^American Society of Anesthesiologists

^d^Total thyroidectomy

^e^Thyroid lobectomy

^f^Central neck dissection

## Discussion

The results of the present study demonstrated that early post-operative US evaluation after IONM-assisted thyroid surgery improves the overall accuracy of IONM alone in assessing laryngeal function after thyroid surgery. Overall accuracy was 91.4% for IONM and 96.5% for US, respectively.

In our series, inaccurate IONM results were registered in 20 cases (8.5%). In 4 out of 7 cases (57%) in which a TT was planned and the procedure was stopped after SSR or LOS after the dissection of the first side, a TT could have been safely performed, avoiding a potential bilateral laryngeal palsy since post-operative FL showed normal vocal cord motility.

Post-operative laryngeal US is easy to perform, unexpansive, and reproducible. Still, in our series, it was not useful in 12 patients (5.3%) because it was inaccurate in 5 cases and could not assess laryngeal motility status in 7 cases.

Our study confirms that subjective dysphonia is not an accurate parameter to assess laryngeal function [1] since in 25% of patients who referred dysphonia FL showed normal vocal cord motility and in one patient without dysphonia FL showed unilateral vocal cord hypomotility.

Overall, these results suggest that IONM and post-operative US do not replace FL, which remains the gold standard for early detection of laryngeal motility changes also in asymptomatic patients.

In our series, age, sex, mean BMI, different BMI cut-offs (≥ 25 kg/m^2^ vs < 25 kg/m^2^, ≥ 30 kg/m^2^ vs < 30 kg/m^2^), smoking habit, presence of hyperthyroidism, cervico-mediastinal extension of the disease, and American Society of Anesthesiologists (ASA) score were not pre-operative risk factors both for inaccurate IONM and post-operative laryngeal US results (Tables 5 and 6). Among pre-operative parameters, a higher Mallampati score results as a risk factor both for inaccurate IONM and US results, suggesting that the patient’s habitus and difficult intubation play a role both in the intraoperative laryngeal function evaluation recorded by IONM results and in the US vocal cord evaluation. Probably in patients with higher Mallampati score the routinary use of video laryngoscopy-assisted intubation could confirm the correct positioning of the electrodes reducing inaccuracy of IONM results.

We designed the present study since there is no ENT unit in our regional referral centre for thyroid surgery recognised by SIUEC (Società Italiana Unitaria di EndocrinoChirurgia – Unitary Italia Society of Endocrine Surgery). The ENT specialist is an external consultant who is not always available. In this setting, our ENT specialist performs a pre-operative FL in all patients; we send to other national referral centres patients with very high risk of bilateral laryngeal nerve palsy (i.e. pre-operative evidence of unilateral laryngeal paralysis in patients requiring TT, recurrent disease contralateral to previous definitive vocal cord paralysis, etc.), all the procedures are IONM-assisted and, independently from the pre-operative diagnosis, when bilateral ILN dissection is planned, we stop the surgical procedure after the dissection of the first side in case of LOS or SSR. We introduced the US laryngeal evaluation on postoperative day 1, aiming to verify if FL could be avoided, adding further information on laryngeal function in patients who underwent IONM-assisted thyroid surgery. We failed to demonstrate that post-operative FL could be avoided using this approach.

On the other hand, we have learnt to use a simple, unexpansive and reproducible technique that has been included in our clinical practice. First, we understand that the laryngeal US is not feasible in all the patients; indeed, we failed to assess the laryngeal motility status in 7 (3%) cases. Similarly, to our findings, previous studies reporting unsuccessful laryngeal US mainly in the elderly (progressive calcification of the thyroid cartilage), male gender (prominence of the thyroid cartilage), taller height, shorter distance from cricoid to neck incision, difficult intubation with secondary tissue oedema, prolonged operative time [17–31]. The use of combinations of frontal and lateral scans [39] and the use of gel pads [40] have been reported as useful tools to improve the effectiveness of US evaluation. On the other hand, it is not simple to compare the results reported since there are many study design assessing feasibility and accuracy of laryngeal US [17–31, 41]. First of all, preoperative US and postoperative laryngeal US appear to differ, as in the latter case, factors such as tissue oedema, scarring, scar dressings, seromas, and the time elapsed since surgery can potentially alter the acoustic window, making it much more challenging to evaluate laryngeal motility [17–31, 41].

We recognise that a limitation of the present study is the lack of data regarding the pre-operative US assessment of the laryngeal motility status, which could lead to the identification of patients at higher risk of failed postoperative laryngeal US. On the other hand, all patients had a pre-operative FL to assess vocal cord motility and pre-operative data regarding US visualisation of the vocal cords would not have changed the surgical strategy. Furthermore, cases of failed post-operative US laryngeal evaluation reflected real-world data underlying that it is not an adequate technique in all patients. Post-operative laryngeal US could not assess laryngeal motility status in 7 cases: 3 males and 4 females with a mean age of 52 years and a mean BMI of 27 kg/m^2^. In our sturdy, high Mallampati score was the only risk factor for inaccurate US results suggesting that patient’s habitus plays a role in the US vocal cord evaluation. Based on our experience, the greatest difficulties in ultrasound visualization of the glottic plane and vocal cords occur in male patients, due to the thicker thyroid cartilage, and in overweight patients, due to the thick subcutaneous adipose tissue. These conditions create a barrier for the penetration of ultrasound waves to greater depths.

Nonetheless, post-operative laryngeal US improves the overall accuracy of IONM results since we observed inaccurate IONM results in 20 cases (8.5%). IONM errors are reported in 3.8–23% of the procedures [14, 15]. In case of LOS or SSR, in absence of objective ILN damage, the possibility of IONM error should be evaluated, and possible common causes are improper type or dosage of muscle relaxants and recording electrode displacement or dislodgement due to repositioning of the head or body during the operation, manipulation of the trachea/larynx, secretions [1]. Moreover, when a recording side problem is suspected, laryngeal palpation is suggested, the presence of the laryngeal twitch in the absence of IONM signal is suggestive of IONM error but the twitch response varies among patients and the interpretation varies among surgeons [15, 42]. For these reasons, the assessment of real-time vocal cord motility during stimulations evaluated by laryngoscopy has been proposed [15, 42] but considered not easy and eventually induced unpredictable trauma. Aiming to use a non-invasive objective method able to assess real-time vocal cord movement as an alternative to the laryngeal palpation during stimulation, the intraoperative US for vocal cord motility evaluation during stimulation has been proposed [15, 42]. In selected cases (patients with pre-operatively documented laryngeal US feasibility assessing vocal cord motility), intraoperative laryngeal US could be included in the troubleshooting algorithm after intraoperative LOS since twitch response and interpretation by palpation vary among patients and surgeons.

Moreover, in patients with pre-operatively documented laryngeal US feasibility assessing vocal cord motility, in absence of any possible doubt on IONM response and in absence of LOS and SSR, postoperative laryngeal US showing normal vocal cord motility could potentially replace routine postoperative FL. Ideally, a video recording of the postoperative laryngeal US showing normal vocal cord motility should be included in the patient’s documentation. In the remaining cases, routine postoperative FL remains the gold standard in our opinion.

We acknowledge that another limitation of the present study could be related to the relatively small number of patients with vocal cord paralysis which limits, the at least in part, the statistical power for identifying risk factors associated with inaccurate IONM and US results. Moreover, surgeon who performed the postoperative laryngeal US known the IONM results and this could, at least in partially, influence the results. Probably, large multicenter blinded studies are needed to better investigate among risk factors for inaccurate IONM and US results. On the other hand, the variability in the operator experience both for US and FL could be a potential limit to compare results of multicentric studies.

We acknowledge that another limitation of the present study could be related to the variable experience of the anesthesiologists that could influence the accuracy of endotracheal tube placement, and they use different protocols (single induction dose of nondepolarising neuromuscular blocking agents or succinylcholine). These variables could be partially responsible for the high rate of inaccurate IONM results reported in our study. On the other hand, to date, we use IONM in all our procedures to ensure a safe thyroidectomy: if it is true that in some cases of the present series, we stopped a planned bilateral ILN dissection for TT after false positive results of IONM, it is also true that we have no bilateral laryngeal nerve palsy, one of the life-threatening rare but possible complications after bilateral ILN dissection.

## Conclusions

In conclusion, early postoperative US evaluation after IONM-assisted thyroid surgery improves the overall accuracy of IONM alone in assessing laryngeal function after thyroid surgery. Nonetheless, IONM results and postoperative US do not replace FL, which remains the gold standard for early detection of laryngeal motility changes in asymptomatic patients.

## Data Availability

The datasets generated and analyzed during the current study are available from the corresponding author on reasonable request.
